# Anthropometric measurements and prevalence of underweight, overweight and obesity in adult Malawians: nationwide population based NCD STEPS survey

**DOI:** 10.11604/pamj.2013.15.108.2622

**Published:** 2013-07-24

**Authors:** Kelias P Msyamboza, Damson Kathyola, Titha Dzowela

**Affiliations:** 1 World Health Organisation, Malawi Country Office, Lilongwe, Malawi; 2 Ministry of Health, Lilongwe, Malawi

**Keywords:** Anthropometric measurements, overweight, obesity, sub-Saharan Africa, Malawi

## Abstract

**Introduction:**

Overweight and obesity are significant causes of increased morbidity and premature mortality from non-communicable diseases, particularly in sub-Saharan Africa, although local high quality population-based data to inform policies and strategies are lacking.

**Methods:**

Using the WHO STEPwise approach to chronic disease risk factor surveillance, population-based nationwide survey was conducted on participants aged 25-64 years in Malawi. A multi-stage cluster sample design and weighting were used to produce a national representative data for that age range.

**Results:**

A total of 4845 participants (65.7% females, 87.6% from rural areas) had complete anthropometric data and included in this analysis. Overall (both sexes) population-based mean body weight, height, systolic blood pressure, diastolic blood pressure, blood glucose and cholesterol were estimated at 58.7 kg, 159.9 cm, 133.4 mmHg, 79.5 mmHg, 4.3 mmol/L, 4.4 mmol/L respectively. Prevalence of underweight, overweight, obesity, overweight and/ or obesity and central adiposity were 6.5%, 17.3%, 4.6%, 21.9% and 28.8% respectively. Overweight, obesity, overweight and/ or obesity and central adiposity were more frequent in females than males (20.7% vs 14.1%, 7.4% vs 2.0%, 28.1% vs 16.1% and 52.8% vs 5.6%), in urban than rural areas (23.2% vs 16.6%, 12.0% vs 3.7%, 35.2% vs 20.2%) respectively.

**Conclusion:**

This study demonstrated that overweight and/ or obesity is the major public health problem affecting at least one in five adults in Malawi. The problem is more frequent in females than males and urban than rural. Implementation of primary health care approaches such as WHO package for essential non-communicable diseases could reduce the problem.

## Introduction

Overweight and obesity are the major risk factors for increased morbidity, disability, and premature mortality from cardiovascular disease (mainly hypertension, heart disease and stroke), type 2 diabetes mellitus; musculoskeletal disorders (osteoarthritis and chronic back pain) and some cancers (endometrial, breast, and colon) [1–4]. It is estimated that the burden of hypertension attributable to obesity is approximately 80% for men and 60% for women; the odds ratio for hypertension is 1.7 for overweight compared with normal weight individuals. Using body mass index (BMI) as an anthropometric measure of adiposity, each 5 units above the overweight category (BMI ≥ 25 kg/m^2^) is associated with approximately 30% higher overall mortality and 40% higher for cardiovascular mortality [5–7]. Once considered as a health problem for high-income countries, overweight and obesity are now on the rise in low- and middle-income countries, particularly in urban settings. In sub-Saharan Africa (SSA), the global epidemic of overweight and obesity - “globesity“ - is rapidly becoming a major public health problem because of uncontrolled rapid urbanisation and changes in lifestyles. It is estimated that 25% to 60% of urban women are overweight, prevalence of hypertension (defined as blood pressure of 140/90 mmHg or more) is on the rise and commonly exceeds 20%-25% in rural areas, 30% in urban and semi-urban areas and prevalence of diabetes of up 16% (range 0% to 16%) have been reported. Type 2 diabetes accounts for over 90% of diabetes in sub-Saharan Africa [8–13].

In Malawi, just like other sub-Saharan countries, recent population-based data suggest that non-communicable diseases and their risk factors are major public health problems with estimates of age-sex-standardised cancer incidence rate per 100, 000 population increasing from 31 to 56, 29 to 69 in males and females respectively in the period 1999-2002 to 2007-2010 [14]. Between July and September 2009, we conducted a nationwide population-based cross-sectional survey using WHO STEPS survey tools to determine the magnitude of chronic non-communicable diseases and their risk factors in Malawi. Prevalences of hypertension, diabetes, overweight and/ or obesity, tobacco smoking and harmful use of alcohol in males and females in adults aged 25-64 years old were 37.2% vs 29.2%, 6.5% vs 4.7%, 18.1 vs 35.4%, 25.9% vs 2.9% and 19.0% vs 2.3% respectively [15, 16]. In this paper, the detailed findings on the anthropometric measurements and prevalences of underweight, overweight and obesity and their correlates are presented.

## Methods

### Study design and sample size

This study was a nationwide population- household- based cross-sectional survey designed according to a WHO STEPwise approach to chronic disease risk factor surveillance [17]. Sample size, calculated using the standard formula, was adjusted for design effect for complex sample design set at 1.5, age-sex estimates in the 25-64 age range (8, 10-year intervals) and a non-response rate of 20%. With these adjustments, the final required sample size was 5,760. It was assumed that the non-response rate would be high because participants may refuse blood testing and/ or adhere to fasting, the latter being required for fasting blood glucose testing.

### Sampling of survey sites, households and eligible participants

Enumeration areas (EAs) were used as survey sites. Administratively, Malawi is divided into twenty-eight districts. In turn, each district is subdivided into smaller administrative units called traditional authorities (TAs). Each TA is sub-divided into EAs by the National Statistical Office (NSO). Enumeration areas are classified as urban or rural. Each EA has demographic data and a sketch map. The sketch map shows the EA boundaries, location of buildings, and other landmarks. The list of all EAs in Malawi from population and housing census conducted in June 2008 was obtained from NSO. This list was used as a sampling frame for the random selection of EAs. According to the WHO NCD STEPS Survey manual Part 2 section 2 [17], in each EA 30-50 households could be selected and in each household only one eligible participant could be selected. We settled for 40 households per EA. Therefore to reach the required sample size, the total number of EAs to be selected was 144 EAs (5760/40). The 144 EAs were randomly selected nationwide using the probability proportional to size (PPS) sampling method. In each EA, 40 households were randomly selected using systematic sampling method. Sampling interval was calculated by dividing the total number of households in the EA as given by the NSO by 40 (the number of households to be selected). At household level, only one eligible participant was selected using the Kish sampling method built-in personal digital assistant (PDA, HP iPAQ). Households with no eligible participant were not replaced.

### Recruitment of participants and data collection

Eligible participants were all adults aged 25-64 years. Participants were involved in the study for two days: day one was for the questionnaire and anthropometric measurements and day two was for blood pressure measurement and laboratory tests. Formal written consent was obtained. Participants with abnormal physical or laboratory findings as defined below were counseled and referred to their nearest health facility for further action and follow up. Body measurements and laboratory tests were performed by nurses and clinical officers while enumerators conducted the interviews. A total of seven survey teams, each with 8 members were deployed to collect data over a period of 30 days between July and September 2009.

### Step 1: demographic and lifestyle data collection

Demographic and lifestyle data were collected using WHO STEPS questionnaire. The questionnaire was programmed on PDA. It consisted of core (age, sex and education in years and current exposure to tobacco and alcohol, diet and physical activity), expanded (rural/urban setting, occupation, average household income) and optional (marital status, medical and health history, past history of smoking and alcohol consumption) variables. The medical and health history component included questions on medication, cigarette use, diabetes mellitus and hypertension. The English questionnaire was translated into two main local languages (Chichewa and Tumbuka).

### Step 2: Anthropometric measurements

Anthropometric measurements that were performed were; body weight, height, waist and hip circumference and blood pressure. Body weight measurements were taken on a pre-calibrated weighing bathroom scale (Seca gmbh & Co., Hamburg, German). The scales were calibrated daily using a known weight (1kg packet of sugar). Participants were weighed dressed in light clothing and barefoot. Measurements were taken to the nearest 0.1 kg. Height was measured with the participant standing upright against a wall on which a height mark was made. Measurements were taken with the participant in barefoot, standing with the back against the wall and head in the Frankfort position with heels together. The participant was asked to stretch to the fullest. After being appropriately positioned, the participant was asked to exhale and a mark with a white chalk was made to mark the height. The height was then measured in centimeters from the mark to the floor using the tape-measure. Measurements were taken to the nearest 0.1 cm. Waist circumference was measured using a tape-measure in centimeters, and the measurement was made in the mid-axillary line midway between the last rib and the superior iliac crest. Measurements were taken to the nearest 0.1 cm. Hip measurement was also made using a tape-measure placed horizontally at the point of maximum circumference over the buttocks. Measurements were taken to the nearest 0.1 cm. Blood pressure measurements were taken using battery powered digital blood pressure machines.(Omron® M4-I, Omron Healthcare Co. Ltd, Hoofddorp, The Netherlands). The participant was asked to sit on the chair and rest quietly for 15 minutes with his/her legs uncrossed. The left arm of the participant was then placed on the table with the palm facing upward.Three readings, 3-5 minutes apart, were then taken on the left arm. During the analysis the average of the last two readings was the final blood pressure reading.

### Step 3: Biochemistry laboratory measurements

On the first day of the survey after step 1 and step 2, participants were asked to starve overnight. Consenting participants were asked not to consume any food except for water after taking supper/dinner of that day until the survey team came again in morning of the following day (day 2). People converged at the agreed place in their community where finger prick blood samples for biochemistry tests were taken. Those that complied with the advice (starving overnight) were eligible for finger prick blood sample collection. Total cholesterol and fasting blood glucose were measured using Accutrend ® Plus machines (Roche, Mannheim, Germany).

### Data management

Data were collected electronically using PDAs programmed with WHO e-STEPS software. There were two sets of PDAs, one set for Step 1 (questionnaire) and Step 2 (anthropometric measurements) and the other set for Step 3 (biochemistry measurements). A total of 50 PDAs were used. Data on the PDAs were downloaded into the computer installed with WHO NCD STEPS software. The files of each participant (questionnaire, anthropometric measurements, biochemistry tests and Kish data) were then merged using the participant identity (PID) number cross-checked with participant name, EA number or village/township name and other particulars where necessary. After merging, common variables in the dataset were matched and inconsistencies were corrected.

Data were weighted by calculating sample weights for all records using the probability of selection at each stage of sampling. Thus, for each participant his/her weight was calculated by multiplying the probability of EA selection, the probability of household selection, the probability of participant selection within the household and age-sex population distribution in Malawi. The participant′s weight was equal to the inverse of this product. Data analysis was conducted using WHO e-STEPs software and Epi Info, version 3.5.1 (Centres for Disease Control and Prevention, Atlanta, Ga). Chi-square tests were used to evaluate differences in proportions and student's t-test for differences in means.

### Definitions

Body mass index (BMI) was defined as body weight (kg) divide by height (m2). Underweight, normal weight, overweight and obesity were defined as by WHO as BMI < 18.5, 18.5-24.9, 25.0-29.9 and ≥ 30.0 respectively. Overweight and/or obesity was defined as BMI ≥ 25. Central obesity was defined as waist hip ratio (WHR) of 0.94 or more for men and 0.80 or more for women. Excessive or harmful use of alcohol was defined as alcohol consumption of 5 or more for men, 4 or more for women standard units of alcohol per day for three or more days in a week. High physical activity was defined as a person reaching any of the following criteria: vigorous-intensity activity on at least 3 days achieving a minimum of at least 1,500 metabolic equivalents (MET)-minutes/week or 7 or more days of any combination of walking, moderate- or vigorous intensity activities achieving a minimum of at least 3,000 MET-minutes per week. Moderate physical activity was defined as a person meeting any of the following: 3 or more days of vigorous-intensity activity of at least 20 minutes per day or 5 or more days of moderate-intensity activity or walking of at least 30 minutes per day or 5 or more days of any combination of walking, moderate- or vigorous intensity activities achieving a minimum of at least 600 MET-minutes per week. Low physical activity was defined as a person not meeting any of the above mentioned physical activities. Hypertension was defined as a diastolic blood pressure of 90 mmHg or more or systolic blood pressure of 140 mmHg or more or currently on medication for hypertension (documented in the health booklet). Diastolic blood pressure ≥ 110 mmHg or systolic ≥ 180 mmHg was considered as severe hypertension. Raised fasting blood glucose was defined as blood glucose level ≥ 7.0 mmol/L or currently on medication for diabetes mellitus (documented in the health booklet). Raised total cholesterol was defined as cholesterol level ≥ 5.0 mmol/L [17, 18]. The detailed methods and materials have also been presented elsewhere [15, 16]. Results were considered statistically significant, p < 0.05 or t < 0.05.

### Ethics statement

Ethical approval was granted by the Malawi National Health Sciences Research and Ethics Committee. Written informed consent was obtained before participants were enrolled in the study using the WHO NCD STEPS survey consent form.

## Results

### Socio-demographic characteristics of participants enrolled in study

A total of 5,451 eligible adults were selected and approached to participate in the survey. Of these, 245 (4.5%) refused/were not available while 5,206 (95.5%) consented and took part in the survey. Of the 5,206 participants that took part in the survey, 183 (3.5%) were pregnant women and were excluded in the analysis for this paper to control for the potential influence of pregnancy on body weight, height and biochemistry measurements. Complete data for anthropometric measurements were available and analysed for 4,845 (93.1%) participants. Of the 4,845 participants with complete anthropometric data, 3,181 (65.7%) were females, 4,242 (87.6%) were from rural areas, 4,156 (85.7%) had primary education or none, 3,541 (73.1%) were married or cohabiting, 530 (10.9%) were tobacco smokers, 639 (13.2%) were alcohol drinkers and 387 (10.1%) had low level of physical activity (Table 1).

**Table 1:** Population-based anthropometric measurements for adult Malawians: National NCD STEPS survey 2009

	Both sexes			Male			Female			Urban			Rural		
	n	mean	95%CI	n	mean	95%CI	n	mean	95%CI	n	mean	95%CI	n	mean	95%CI
**Body weight (kg)**	4845	58.7	58.2-59.1	1664	60.4*	60.0-60.9	3181	56.8	56.2-57.4	603	62.7*	60.9-64.6	4242	58.1	57.7-58.5
**Body height (cm)**	4845	159.9	159.5-160.3	1664	164.3*	163.8-164.8	3181	155.4	155.0-155.7	603	160.8	159.9-161.7	4242	159.8	159.4-160.2
**Body mass index (Kg/m** ^**2**^ **)**	4845	23.0	22.8-23.1	1664	22.4	22.3-22.6	3181	23.5*	23.3-23.7	603	24.4*	23.7-25.0	4242	22.8	22.6-22.9
**Waist circumference (cm)**	4734	77.9	77.3-78.6	1639	77.3	76.6-77.9	3095	78.6	77.8-79.4	591	81.5	79.2-83.8	4143	77.5	76.8-78.1
**Hip circumference (cm)**	4733	91.1	90.3-91.8	1638	89.9	89.2-90.7	3095	92.2*	91.3-93.2	591	95.8*	92.8-98.8	4142	90.4	89.7-91.2
**Systolic blood pressure (mmHg)**	3658	133.4	132.4-134.5	1172	135.8*	134.4-137.1	2486	131.3	130.1-132.5	377	131.5	127.9-135.2	3281	133.6	132.5-134.7
**Diastolic blood pressure (mmHg)**	3658	79.5	78.8-80.1	1172	79.1	78.3-79.9	2486	79.8	79.1-80.5	377	80.5	78.5-82.6	3281	79.4	78.7-80.1
**Heart rate reading 1-3 (beats per minute)**	3658	76.2	75.3-77.1	1172	70.8	69.7-72.0	2486	81.2*	80.3-82.0	377	76.0	74.0-78.0	3281	76.2	75.3-77.2
**Blood glucose (mmol/l)**	2865	4.3	4.2-4.4	900	4.3	4.2-4.5	1965	4.2	4.1-4.4	339	4.3	4.1-4.5	2526	4.3	4.1-4.4
**Blood cholesterol (mmol/l)**	2433	4.4	4.3-4.4	768	4.3	4.3-4.4	1665	4.4	4.4-4.5	272	4.5	4.4-4.7	2161	4.3	4.3-4.4

Key: CI= confidence interval, n= number of participants in the group

* statistically significant males vs females, urban vs rural, *t* <0.05

### Anthropometric measurements

Mean systolic blood pressure, body weight and height were higher in males than females (135.8 mmHg vs 131.8 mmHg, 60.4 Kg vs 56.8 Kg and 164.3 cm vs 155.4 cm respectively, all t < 0.05). Mean body mass index, hip circumference and heart rate were higher in females than males (23.5 Kg/m^2^ vs 22.4 Kg/m^2^, 92.2 cm vs 89.9 cm and 81.2 beats/minute vs 70.8 beats/minutes respectively, all t < 0.05). By urban/rural, mean body weight, body mass index and hip circumference were higher in urban than rural (62.7 Kg vs 58.1 kg, 24.2 kg/m^2^ vs 22.8 kg/m^2^, 95.8 cm vs 90.4 cm respectively, all t <0.05). No significant differences were observed in males vs females, urban vs rural in mean diastolic blood pressure, blood glucose and blood cholesterol. Overall (both sexes) population mean body weight, height, systolic blood pressure, diastolic blood pressure, blood glucose and cholesterol were estimated at 58.7 kg, 159.9 cm, 133.4 mmHg, 79.5 mmHg, 4.3 mmol/L, 4.4 mmol/L respectively (Table 1).

### Prevalence of underweight, overweight and obesity

Population-based prevalence of underweight, overweight, obesity, overweight and/ or obesity and central adiposity in adults aged 25-64 years old were 6.5%, 17.3%, 4.6%, 21.9%, 28.8% respectively. In univariate analysis, overweight, obesity, overweight and/ or obesity and central adiposity were more frequent in females than males (20.7% vs 14.1%, 7.4% vs 2.0%, 28.1% vs 16.1% and 52.8% vs 5.6%), in urban than rural (23.2% vs 16.6%, 12.0% vs 3.7%, 35.2% vs 20.2%) respectively, all t < 0.05. There were no significant differences observed in underweight in males vs females, urban vs rural and central adiposity in urban vs rural (Table 2).

**Table 2:** Population-based prevalence of underweight, overweight and/ or obesity and central adiposity in adult Malawians: National NCD STEPS survey 2009

	Both sexes			Male			Female			Urban			Rural		
	n	%	95%CI	n	%	95%CI	n	%	95%CI	n	%	95%CI	n	%	95%CI
Underweight (BMI <18.5 kg/m^2^)	4845	6.5	5.8-7.3	1664	6.2	5.0-7.7	3181	6.9	6.0-7.8	603	5.9	3.8-9.1	4242	6.6	5.8-7.5
Normal weight (BMI 18.5-24.9 kg/m^2^)	4845	71.5	69.9-73.0	1664	77.8	75.6-79.8	3181	65.1	62.9-67.1	603	58.9	52.5-65.0	4242	73.1	71.6-74.7
Overweight (BMI 25.0-29.9 kg/m^2^)	4845	17.3	16.1-18.7	1664	14.1	12.4-16.0	3181	20.7*	19.0-22.5	603	23.2*	18.9-28.2	4242	16.6	15.3-18.0
Obese (BMI ≥30 kg/m^2^)	4845	4.6	4.0-5.3	1664	2.0	1.4-2.7	3181	7.4*	6.4-8.5	603	12.0*	8.8-16.2	4242	3.7	3.1-4.3
Overweight and/ or obese (BMI ≥25.0 kg/m^2^)	4845	21.9	20.4-23.5	1664	16.1	14.2-18.1	3181	28.1*	26.0-30.2	603	35.2*	29.2-41.7	4242	20.2	18.7-21.9
Central adiposity (WHR men ≥ 0.95, women ≥ 0.85)	4845	28.8	27.2-30.4	1664	5.6	4.4-7.0	3181	52.8*	50.5-55.1	603	30.0	26.2-34.1	4242	28.6	26.9-30.4

BMI= body mass index, CI= confidence interval, n= number of participants in the group, WHR= waist hip circumference ration

* statistically significant males vs female, urban vs rural *p* <0.05

### Factors associated with overweight and/ or obesity (BMI ≥ 25 kg/m^2^)

In uni-variate analysis, female gender, urban dwelling, not smoking tobacco, high blood pressure, high fasting blood glucose and high blood cholesterol were significantly associated with overweight and/ or obesity. Overweight and/ or obesity were more frequent in those with low than those with moderate or high levels of physical (24.6% vs 21.7%) but the association was not statistically significant (Table 3). In multi-variate analysis (logistic regression), female gender, urban dwelling, age 35-44 years old, currently married or cohabiting, not smoking tobacco, high blood pressure and high blood cholesterol were important factors/conditions associated with overweight and/ or obesity (Table 3).

**Table 3:** Population-based Risk factors for overweight and/ or obesity in adult Malawians: National NCD STEPS survey 2009

	n	%Overweight and/ or obesity (BMI ≥25.0 kg/m^2^)	95%CI
**Gender**			
Female	3181	28.1*	26.0-30.2
Male	1664	16.1	14.2-18.1
Both sexes	4845	21.9	20.4-23.5
**Age group (years)**			
25-34	2094	20.2	18.5-21.9
35-44	1244	24.4	21.7-27.2
45-54	879	22.7	19.8-26.0
55-64	628	22.3	18.7-26.4
25-64	4845	21.9	20.4-23.5
**Residence**			
Urban	603	35.2*	29.2-41.7
Rural	4242	20.2	18.7-21.9
**Education attained**			
Primary (standard 1-8) or none	4156	21.5	19.9-23.1
Secondary or above	686	24.4	20.8-28.5
**Marital status**			
Currently married or cohabitating	3541	22.1	20.4-23.9
Divorced/separated or widowed	1296	21.4	19.1-24.0
**Smoking**			
Non-smokers	4315	24.0*	22.4-25.6
Current smokers	530	10.2	7.9-13.1
**Alcohol**			
Non-drinkers	4206	22.9*	21.3-24.7
Current drinkers	639	17.3	14.1-21.1
**Physical activity**			
Low level	387	24.6	20.5-29.2
Moderate or high level	3430	21.7	20.0-23.4
**Blood pressure**			
Normal	2485	19.3	17.6-21.1
Raised blood pressure (SBP ≥ 140 or DBP ≥ 90) or currently on medication for hypertension	1173	27.6	24.7-30.8
**Fasting blood glucose**			
Normal (<7.0 mmol/L)	2840	22.3	20.4-24.3
Raised (≥ 7.0 mmol/L)	25	41.8	22.8-63.5
**Total cholesterol**			
Normal (<5.0 mmol/L)	2195	22.7	20.5-25.0
Raised (≥ 5.0 mmol/L)	238	35.1	28.9-42.0

Key: BMI= body mass index, CI= confidence interval, M= meter, n= number of participants in the group

* statistically significant *p* <0.05

## Discussion

High prevalence of overweight and/or obesity (BMI ≥ 25kg/m^2^) of one in five and relatively low prevalence of underweight/under nutrition (BMI≤ 18.5kg/m^2^) of less than 7% in adult Malawians, the inverse of what was previously reported, is the most striking feature of this study. Population-based anthropometric studies conducted in Malawi in 1990s and early 2000, reported low prevalence of overweight of 0%- 6% but high prevalence of underweight of up to 40.9% (50.0% in males and 35.7% in females) demonstrating that under nutrition not overweight was major public health problem in adults then [19–21]. In adults, overweight/obesity increases with increasing age and therefore it is unlikely that differences in age group of study participants (25-64 years in this study, 45-94 years in previous studies) is the cause of the shift [1, 2, 22]. Nutrition transition, changes in lifestyles, rapid uncontrolled urbanisation, increasing income, consumption of high-fat food accompanied by decreasing physical activity are the causes of overweight/obesity epidemic in SSA [5, 10, 22–23]. This study confirmed that Malawi is one the countries that experienced epidemiological transition and overweight/obesity is now a major public health problem affecting 22% of adult population, with the epidemic being more common in females than males, urban than rural areas (28.1% vs 16.1%, 35.2% vs 20.2% respectively). The Overweight/obesity epidemic, with prevalence of obesity as high as 42% among urban women, have been reported from other countries in SSA [24–29].

Our findings of female gender, urban residence and age as significant risk factors for overweight/obesity and that overweight/obesity is associated with hypertension were consistent with findings from other studies [23–29]. However, this study is one of the few studies (if any) in sub-Saharan Africa to document the association between BMI and tobacco and alcohol use. Overweight/obesity was more frequent in non- tobacco smokers than smokers (24.0% vs 10.2%), non- alcohol drinkers than drinkers (22.9% vs 17.3%) consistent with findings from other studies that tobacco use is associated with low BMI [30]. All forms of tobacco produce free radicals that deplete antioxidants like Vitamin C, E and carotenoids and cause oxidative damage to DNA, proteins and lipids. Tobacco use also impairs the immune system, making tobacco users more susceptible to infectious agents. The interactions between oxidative stress and infections caused by tobacco use could explain why tobacco use is associated with low BMI [30]. In this population, level of education attained, physical activity, blood cholesterol and fasting blood glucose were associated with overweight/obesity but the associations were not statistically significant.

Observations from this study that adult Malawian males were taller, heavier and had higher systolic blood pressure than females, that females had higher body mass index, hip circumference and heart rate than males, and that adults in urban areas were heavier, had higher body mass index and hip circumference than in rural areas were also consistent with findings from previous population-based studies in Malawi and SSA region [19–21, 24–29]. Comparing our findings to available data collected in 1996, it could be suggested that from 1996 to 2009, in males, mean body weight increased from 54.1 kg to 60.4 kg, BMI from 19.7 kg/m^2^ to 22.4 kg m^2^ while mean height reduced from 165.8 cm to 164.3 cm. Prevalence of overweight/obesity (BMI ≥ 25.0 kg/m^2^) increased from 0.0% to 16.1% while underweight (BMI ^2^) reduced from 36.1% to 6.2%. In females, mean body weight increased from 49.0 kg to 56.8 kg, height from 155.2 cm to 160.8 cm and BMI from 20.5 kg/m^2^ to 23.5 kg/m^2^. Prevalence of overweight/ obesity increased from 6.4% to 28.1% while underweight reduced from 27.0% to 6.9% [19]. This demonstrated that epidemiological anthropometric transition occurred in Malawi just like in other sub-Saharan countries.

Documentation of the existence of overweight/obesity epidemic and its risk factors in Malawi calls for development and implementation of evidence-informed policies, strategies and interventions for resource and community mobilisation. The WHO primary health care approach for prevention and control of NCDs- WHO package for essential non-communicable diseases (WHO- PEN) could be used as one of the strategies to contain the epidemic of NCDs and their risk factors [31]. High out-patient department (OPD) utilisation rate of 1,288 visits per 1, 000 population in public health facilities and the existing of well structured and utilised community out- reach clinics could be used as an opportunity for implementing WHO PEN [32].

In SSA, large body shape (overweight/obesity) is perceived as being rich in males, sexually attractive in females and healthy- interpreting fatness as a sign of good health and absence of disease [33, 34]. This misconception emphasises the need of taking into account gender and socio-cultural issues when developing and implementing evidence-informed lifestyle health promotion interventions. Change is more likely to happen if/when the concerned individual understands that there is a problem that needs changing [35].

### Limitations of the study

Over-representation of females was one of the limitations of this study; two thirds of participants were females. However, it was unlikely that this had an influence on the results for women because data were weighted (standardised) for age and sex to national population. The over representation of females was not by study design because at household level, eligible participants were randomly selected using the Kish sampling method built-in the PDAs after entering their name, sex and age. Refusals/non-availability (though relatively small (245 (5%) out of 5,451 eligible participants), was another limitation of this study. Specifically, males aged 25 - 34 years were the ones that were under-represented based on 2008 National Statistical Office Population figures (42.5% vs 47.5%, p36].

## Conclusion

This study demonstrated that overweight and obesity were the major public health problems in Malawi affecting one in five adults (overall), one in three women and one in three people in urban areas. Risk factors and the need for taking into account gender and socio-cultural issues when developing and implementing evidence-informed strategies and interventions for lifestyle health promotion have been highlighted. Implementation of WHO package for essential non-communicable diseases could prevent and control overweight.
